# Cysteine dioxygenase and taurine are essential for embryo implantation by involving in E_2_-ERα and P_4_-PR signaling in mouse

**DOI:** 10.1186/s40104-022-00804-1

**Published:** 2023-01-05

**Authors:** Di Zhang, Zhijuan Wang, Xuan Luo, Hongzhou Guo, Guobin Qiu, Yuneng Gong, Hongxu Gao, Sheng Cui

**Affiliations:** 1 College of Veterinary Medicine, Yangzhou University, Yangzhou, Jiangsu 225009 People’s Republic of China; 2 Jiangsu Co-Innovation Center for Prevention and Control of Important Animal Infectious Diseases and Zoonoses, Yangzhou University, Yangzhou, 225009 People’s Republic of China; 3 State Key Laboratory of Agrobiotechnology, College of Biological Sciences, China Agricultural University, 100193 Beijing, People’s Republic of China; 4 Institute of Reproduction and Metabolism, Yangzhou University, 225009 Jiangsu, People’s Republic of China

**Keywords:** CDO, E_2_, Embryo implantation, P_4_, Taurine

## Abstract

**Background:**

Taurine performs multiple physiological functions, and the maintenance of taurine level for most mammals relies on active uptake from diet and endogenous taurine synthesis through its synthesis enzymes, including cysteine dioxygenase (CDO). In addition, uterus tissue and uterus fluid are rich in taurine, and taurine synthesis is regulated by estrogen (E_2_) and progesterone (P_4_), the key hormones priming embryo-uterine crosstalk during embryo implantation, but the functional interactions and mechanisms among which are largely unknown. The present study was thus proposed to identify the effects of CDO and taurine on embryo implantation and related mechanisms by using *Cdo* knockout (KO) and ovariectomy (OVX) mouse models.

**Results:**

The uterine CDO expression was assayed from the first day of plugging (d 1) to d 8 and the results showed that CDO expression level increased from d 1 to d 4, followed by a significant decline on d 5 and persisted to d 8, which was highly correlated with serum and uterine taurine levels, and serum P_4_ concentration. Next, *Cdo* KO mouse was established by CRISPER/Cas9. It was showed that *Cdo* deletion sharply decreased the taurine levels both in serum and uterus tissue, causing implantation defects and severe subfertility. However, the implantation defects in *Cdo* KO mice were partly rescued by the taurine supplementation. In addition, *Cdo* deletion led to a sharp decrease in the expressions of P_4_ receptor (PR) and its responsive genes *Ihh*, *Hoxa10* and *Hand2*. Although the expression of uterine estrogen receptor (ERα) had no significant change, the levels of ERα induced genes (*Muc1*, *Ltf*) during the implantation window were upregulated after *Cdo* deletion. These accompanied by the suppression of stroma cell proliferation. Meanwhile, E_2_ inhibited CDO expression through ERα and P_4_ upregulated CDO expression through PR.

**Conclusion:**

The present study firstly demonstrates that taurine and CDO play prominent roles in uterine receptivity and embryo implantation by involving in E_2_-ERα and P_4_-PR signaling. These are crucial for our understanding the mechanism of embryo implantation, and infer that taurine is a potential agent for improving reproductive efficiency of livestock industry and reproductive medicine.

**Supplementary Information:**

The online version contains supplementary material available at 10.1186/s40104-022-00804-1.

## Background

High rate of embryo loss in early pregnancy is a major constraint both in livestock industry and human reproduction, whereas much pregnancy wastage is caused by the failure of embryo implantation [1–3]. The implantation of the blastocyst into the maternal uterus is a crucial step in mammalian reproduction [4, 5]. It is generally accepted that embryo implantation depends on blastocyst quality, endometrial receptivity, and the synchronization of their development [6]. In mice, embryo implantation consists of apposition between the trophectoderm layer of blastocyst and the luminal epithelium (LE), attachment and final invasion into the LE [7]. Upon embryo invasion, the uterine stromal cells are rapidly remodeled in the process of decidualization, which is characterized by morphological and functional changes in stromal cells in the form of proliferation and differentiation into large epithelioid decidual cells [8]. However, the cell proliferation and differentiation of specific uterine cell types in early pregnancy are dependent on the coordinated actions of ovarian steroid hormones, including progesterone (P_4_) and estrogen (E_2_). During mouse pregnancy, an E_2_ surge on d 1 stimulates uterine epithelial cell proliferation, and the decline of E_2_ level on d 2 leads to apoptosis of a large number of epithelial cells. P_4_, from the newly formed corpora lutea on d 3 [8], initiates uterine stromal cell proliferation. In conjunction with P_4_, an acute E_2_ spike on d 4 further stimulates uterine stromal cell proliferation and renders the uterus receptivity for the blastocyst to implant [8–10]. However, it is still largely unknown about the molecular mechanisms of coordinate proliferative events induced by E_2_ and differentiative processes directed by P_4._

Although the receptivity of the uterus during implantation is primed by E_2_ and P_4_, their actions on cell proliferation and differentiation are complicated, and the relative molecular mechanisms have been extensively studied. The related molecular and genetic studies indicate that E_2_ and P_4_ act respectively via estrogen receptor 1 (*Esr1*, ERα) [11] and progesterone receptor (*Pgr*, PR) [12] to govern the embryo-uterine crosstalk during peri-implantation stage by targeting local transcriptional factors, signals or paracrine molecules [13], some of which include Muc1 [14], Ltf [15], Hox10a [16, 17], Hand2 [17] and IHH [18, 19]. In addition, uterine epithelium and its secretions are essential for uterine receptivity and embryo implantation. Uterine epithelium includes LE and glandular epithelium (GE), which directly synthesize, secrete or selectively transport a wide variety of substances from serum and transudate, collectively termed histotroph [20], into uterus lumen. Whereas histotroph is complex and comprised of many different substances, such as leukemia inhibitory factor (LIF), ions, sugars, lipids, proteins and amino acids [20–24], among which taurine is included [25]. It is much interested that taurine concentration in the uterine luminal fluid (UFL) of mouse is much higher during the implantation [25]. But as we know, there is no direct evidence about the functional relations of taurine with uterine receptivity and embryo implantation, although it is reported that *Cdo* knockout (KO) mice exhibit impaired reproductive capacity [26].

Taurine is one of the most abundant non-essential amino acids in most mammals [27], and performs numerous physiological functions, including bile salts synthesis and hepatoprotection [28], energy metabolism [29], maintenance of Ca^2+^ homeostasis [30], anti-oxidative, osmoregulation, anti-inflammatory and anti-apoptotic [31–33]. While the maintenance of the body taurine level mainly relays active uptake from diet and the endogenous taurine synthesis through the sequential actions of its synthesis enzymes, including cysteine dioxygenase (CDO) [34].

CDO is a critical enzyme for taurine synthesis and CDO expression has been detected in liver, adipose tissue, pancreas, kidneys, lungs and reproductive system [35]. *Cdo* KO results in a higher incidence of postnatal mortality, retards postnatal growth and damages male fertility [26, 36]. In addition, CDO is highly expressed in mouse ovary and uterus [37], but the functions of CDO in female reproduction remains unclear. Interestingly, uterus tissue and ULF are rich in taurine, and ULF taurine concentration is increased during embryo implantation [25]. Furthermore, CDO expression in uterus is up-regulated by P_4_, whereas E_2_ decreases CDO expression [37]. In addition, our recent study shows that E_2_ regulates taurine synthesis through E_2_-ERα-CSD signaling [38]. These make us to hypothesize that CDO may play important roles in E_2_ and P_4_ primed embryo implantation, possibly through the physiological actions of taurine. The present study was thus proposed to identify the effects of CDO and taurine on embryo implantation and illustrate the related mechanisms by using *Cdo* KO and ovariectomy (OVX) mouse models.

## Materials and methods

### Animals and treatments

Eight weeks old ICR mice were used in fertility test and other animal experiments. *Cdo* KO mice were generated by using 129 mice (Additional file 1). Mice were raised in controlled temperature (25 ± 1 °C) and humidity (60%–70%) with a 12 h light, 12 h dark cycle. The animal experiments were approved by the Chinese Association for Laboratory Animal Sciences. Virgin female mice were mated with sexually matured males to induce pregnancy (day 1 is the day of vaginal plug checked, d 1). Embryos were collected from oviducts on d 1. The implantation sites (IS) were visualized by intravenous injection of 0.1 mL 1% Chicago blue dye (Sangon Biotech, Shanghai, China) in saline on d 5 [39]. To investigate the ovarian hormonal influence on uterine CDO expression, wild-type (WT) mice were ovariectomized. Oil, 100 ng/mouse 17β-estradiol (E_2_; MedChemExpress, NJ, USA) or 2 mg/mouse progesterone (P_4_; MedChemExpress, NJ, USA) was injected 7 d later [40]. The treated mice were then sacrificed at indicated times for further experiments.

### Real-time quantitative PCR (RT-qPCR) and common PCR

Total RNA of the uterus tissues was isolated using the TRIzol reagent (Takara, Dalian, China), purified by DNase I and quantified by spectrophotometry. 1 μg purified total RNA was used as a template for cDNA synthesis using HiScript Reverse Transcriptase (Vazyme, Nanjing, China) according to the manufacturer’s instructions. RT-qPCR was performed using SYBR Green master mix (Vazyme, Nanjing, China) in the StepOnePlus Real-Time PCR System (Applied Biosystems, Foster City, CA, USA) and reactions were done in triplicate. RT-qPCR conditions were as follows: 95 °C for 2 min, followed by 40 cycles of 95 °C for 15 s and 60 °C for 1 min. Relative gene expressions were normalized to endogenous control *Gapdh*. All Primers listed in Table S3 were designed using NCBI.

The genotype identification of the *Cdo* KO mice was performed by common PCR using primers as described in Table S4. Amplifications were carried out on PCR instrument (Bio-Rad, Hercules, CA, USA) using the following protocol: 94 °C for 5 min (one time); 94 °C for 50 s, 65 °C for 30 s, 72 °C for 30 s (35 times); 72 °C for 10 min; and holding at 4 °C.

### Western blotting (WB)

The uterus tissues were lysed with RIPA buffer (Beyotime, Shanghai, China) containing 1 mmol/L phenylmethanesulfonyl fluoride (PMSF, Sangon Biotech, Shanghai, China). The protein concentration of each group was determined by using the BCA assay reagent (CoWin Biosciences, Jiangsu, China) according to the manufacturer’s recommendations. Equal amounts of 50 μg proteins were electrophoresed on 12% sodium dodecyl sulfate–polyacrylamide gel (SDS-PAGE), and the bands were transferred to 0.45 μm polyvinylidene difluoride (PVDF) membrane (Millipore, MA, USA). The membrane was blocked with 5% (w/v) nonfat dry milk in 0.05 mol/L pH 7.4 Tris buffered saline (TBS) for 3 h and incubated with CDO antibody (ab53436, abcam, Cambridge, UK; 1:2000), internal control GAPDH antibody (AM4300, Ambion, TX, USA; 1:10,000), PR antibody (ab2765, abcam, Cambridge, UK; 1:2000) or ERα antibody (ab32063, abcam, Cambridge, UK; 1:2000) overnight at 4 °C. The PVDF membrane was then washed 3 times for 30 min in TBST (0.1% Tween-20 in TBS) and incubated for 2 h with horseradish peroxidase-conjugated goat anti-rabbit IgG or horseradish peroxidase-conjugated goat anti-mouse IgG (Zhongshan, Beijing, China). After washing for 30 min with 3 changes of TBST, the membrane was treated with the ECL kit (Vazyme, Nanjing, China) and visualized by Tannon gel imager (Tanon, Shanghai, China). The intensity values pertaining to each group were normalized against the optical density of GAPDH corresponding to the same group.

### Immunohistochemistry (IHC) and immunofluorescence (IF)

Tissues were fixed in 4% paraformaldehyde, dehydrated via graded ethanol solutions, and then embedded in paraffin to obtain 5 μm thick sections. IHC was performed as previously described [41]. The sections were incubated with CDO antibody (ab53436, abcam, Cambridge, UK; 1:200), PR antibody (ab2765, abcam, Cambridge, UK; 1:200), ERα antibody (ab32063, abcam, Cambridge, UK; 1:200) or Ki67 antibody (D385, CST, MA, USA; 1:200) diluted in PBS overnight at 4 °C. After washing with PBS for 30 min, the sections were incubated with biotinylated goat anti-rabbit/mouse IgG (31820/31802, Thermofisher, Waltham, MA, USA; 1:200) for 3 h at RT. After washing with PBS for 30 min, the sections were incubated with streptavidin peroxidase complex (SA10001, Thermofisher, Waltham, MA, USA; 1:200) for 30 min at room temperature. Finally, the signals were visualized by incubating the sections with 0.05 mol/L Tris–HCL (pH 6.5) containing 0.06% (w/v) diaminobenzidine (DAB, ZSGB-Bio, Beijing, China) and 0.03% (v/v) H_2_O_2_. For IF staining, Hand2 (ab200040, abcam, Cambridge, UK; 1:200), Muc1 (ab15481, abcam, Cambridge, UK; 1:200) primary antibodies, and their respective secondary antibodies (Jackson Immuno Research, Philadelphia, PA, USA; 1:200) were used. Nuclear staining was performed using 4’,6-diamidino-2-phenylindole (DAPI) dye (0.1 μg/mL, Beyotime, Shanghai, China). The signals were captured using a microscope (Olympus, Tokyo, Japan).

### In vivo production of embryo

Non-superovulated virgin female mice (8–9 weeks) were mated with adult males. The males and females (1:2) were housed overnight and the presence of a vaginal plug in the following morning was regarded as successful mating (d 1 is the day of vaginal plug checked). Mice were killed by cervical dislocation and oviducts were immediately dissected and placed in M2 solution (CaCl_2_·2H_2_O 1.71 mmol/L, Glucose 5.56 mmol/L, HEPES 20.85 mmol/L, KCl 4.78 mmol/L, MgSO_4_·7H_2_O 1.19 mmol/L, NaCl 94.66 mmol/L, NaHCO_3_ 4.15 mmol/L, Sodium lactate 23.28 mmol/L, Sodium pyruvate 0.33 mmol/L) at 11:00 on d 2. Embryos punched out from the ampulla were digested with hyaluronidase (HA) followed by three times washing with M2 and cultured in KSOM at 37 °C until the time of embryo transfer.

### Embryo transfer

The uterine horn was exposed by flank laparotomy and six expanding embryos were transferred with a minimal amount of medium into the uterine cavity of pseudo-pregnant (d 3) recipients. The uterine horn was then placed back into the abdominal cavity and the incision site was closed. The procedure was repeated in the opposite flank where another six expanding embryos were transferred. The recipients were then placed individually in clean cages to recover from anesthesia in a warm room (28–30 °C).

### Fertility test

*Cdo* KO females and their nesting WT females were caged with adult wild-type males (8–9 weeks) at the rate of male:female = 1:2. Vaginal plug was examined the next morning. Female mice with vaginal plug withdrew from the experiment, while the none plugged females were backed to the test after one day’s rest. The plugged females were caged alone to observe their pregnancy and parturition.

### Measurement of taurine

Uterus, liver, and serum taurine contents were measured by HPLC–UV (HPLC). Firstly, samples were weighed, homogenized and deproteinized using 0.2 mol/L sulfosalicylic acid. After being centrifuged at 14,000 × *g* for 20 min, the supernatants were added into a dual-bed column containing cation exchange resins to remove other amino acids and metabolic precursors of taurine. Secondly, samples were added with 100 μmol/L glutamine as an internal standard. All samples were then filtrated through a 0.22-μm PVDF membrane and saved in −80 °C refrigerator until use. The samples and the standard samples of taurine which were 100, 50, 25, 10, 5, 2 and 1 μmol/L were derivated with OPA (Sigma-Aldrich, St. Louis, MO, USA) solution (20 mg OPA, 2 mL methanol, 80 μL 2-hydroxy-1-etanethiol, 18 mL 0.1 mol/L borate buffer (pH 9.6)) for 3 min. Then 20 μL sample was automatically injected into a six-port valve to analysis with Waters Symmetry C18 Column (4.6 μm, 150 mm × 5 mm) (Waters, Milford, MA, USA) on a Shimadzu HPLC system (Shimadzu, Kyoto, Japan). The HPLC conditions were: flow A: 100% methanol, flow B: sodium phosphate buffer pH 4.7 containing 50% methanol. Flow rate was 1.2 mL/min, and the detection wavelength was 340 nm. The duration times were 2.3 min and 4.95 min for the internal standard and taurine.

### Radioimmunoassay (RIA)

Serum P_4_ and E_2_ were analyzed using RIA reagents provided by the Beijing North Institute Biological Technology (Beijing, China). The minimum detectable concentrations were 2 pg/mL for E_2_ and 0.2 ng/mL for P_4_. For each RIA the intra and inter assay coefficients of variation were respectively less than 15% and 10%.

### Statistical analysis

Statistical analysis was performed using GraphPad Prism 6.0. Data from at least three independent samples were expressed as mean ± SEM. Two group comparison studies were performed using Student’s *t*-test and one-way analysis of variance (ANOVA) for data comprising three or more groups. *P* < 0.05 was considered to be statistically significant.

## Results

### Uterine CDO expression and taurine levels during early pregnancy

**Fig. 1:**
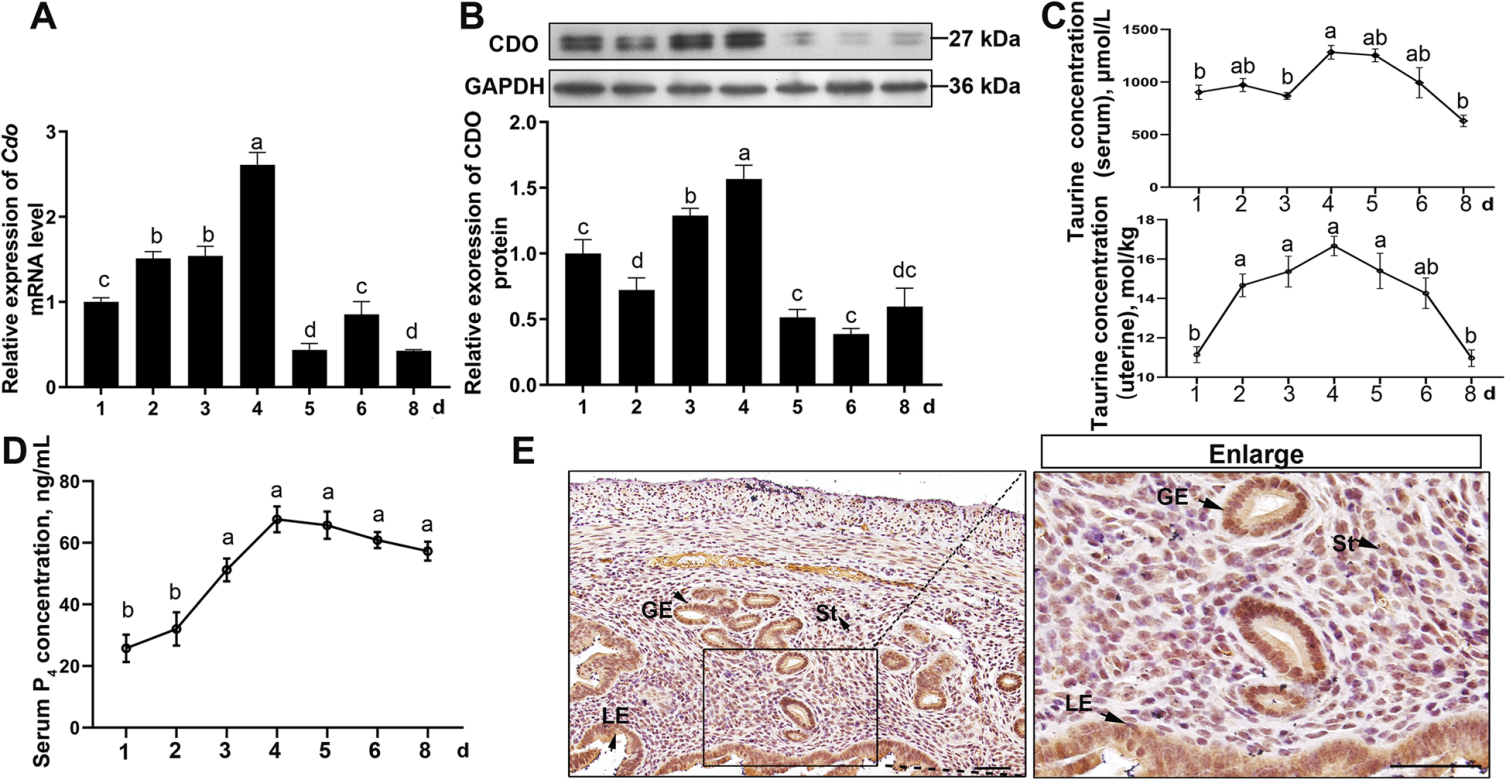
Uterine CDO mRNA and protein expressions and their relations to taurine levels in uterus from d 1 to d 8 of pregnancy mice. **A**, *Cdo* mRNA expression in mouse uteri detected by RT-qPCR (*n* = 3). **B**, CDO protein expression in uteri of d 1 to d 8 pregnancy mice assayed by WB. **C**, Serum and uterine taurine concentrations assayed by HPLC (*n* = 3). **D**, Serum P_4_ concentrations assayed by RIA (*n* = 5). **E**, CDO IHC staining of uterine cross-sections. Data shown as Mean ± SEM. Different letters represent significant differences (*P* < 0.05). d, days post coitum; E_2_, estrogen; GE, glandular epithelium; LE, luminal epithelium; P_4_, progesterone; St, stromal cells. Bars: 50 μm

In order to evaluate the physiological significance of CDO and taurine in adult female mouse, we firstly examined the CDO expression profiles in different organs by RT-qPCR and WB. The results showed that CDO mRNA and protein were highly expressed in mouse uterus (Fig. S1). Next, the uterine CDO mRNA and protein expressions, their relations to the changes of uterine taurine concentrations and serum steroid hormones were analyzed in the duration of early pregnancy, from d 1 to d 8. RT-qPCR results showed that *Cdo* mRNA increased from d 1 to d 4 and reached the maximum on d 4, which followed by a sharp decline on d 5 and persisted on d 6 and d 8 (Fig. 1 A). WB results showed that CDO protein levels were highly correlated with *Cdo* mRNA levels in the duration examined (Fig. 1 B). In addition, IHC staining revealed that CDO was located in uterine epithelial cells, including LE and GE, and some stromal cells (Fig. 1 E). The taurine concentrations in uterus tissue and serum were assayed by HPLC and the results indicated that the uterine taurine levels exhibited an increasing tendency from d 1 to d 4, and reached the maximum on d 4, followed by a steady decline and returned to the similar level of d 1 on d 8 (Fig. 1 C). The serum taurine level also increased from d 1 to d 4, which significantly declined from d 5 to d 8 (Fig. 1 C). In addition, the dynamic patterns of the uterine CDO expression and taurine concentration corresponded to the change of serum P_4_ from d 1 to d 6 (Fig. 1 D). These suggest that CDO might play important roles in regulating embryo implantation.

### Establishment of *Cdo* KO mouse

**Fig. 2:**
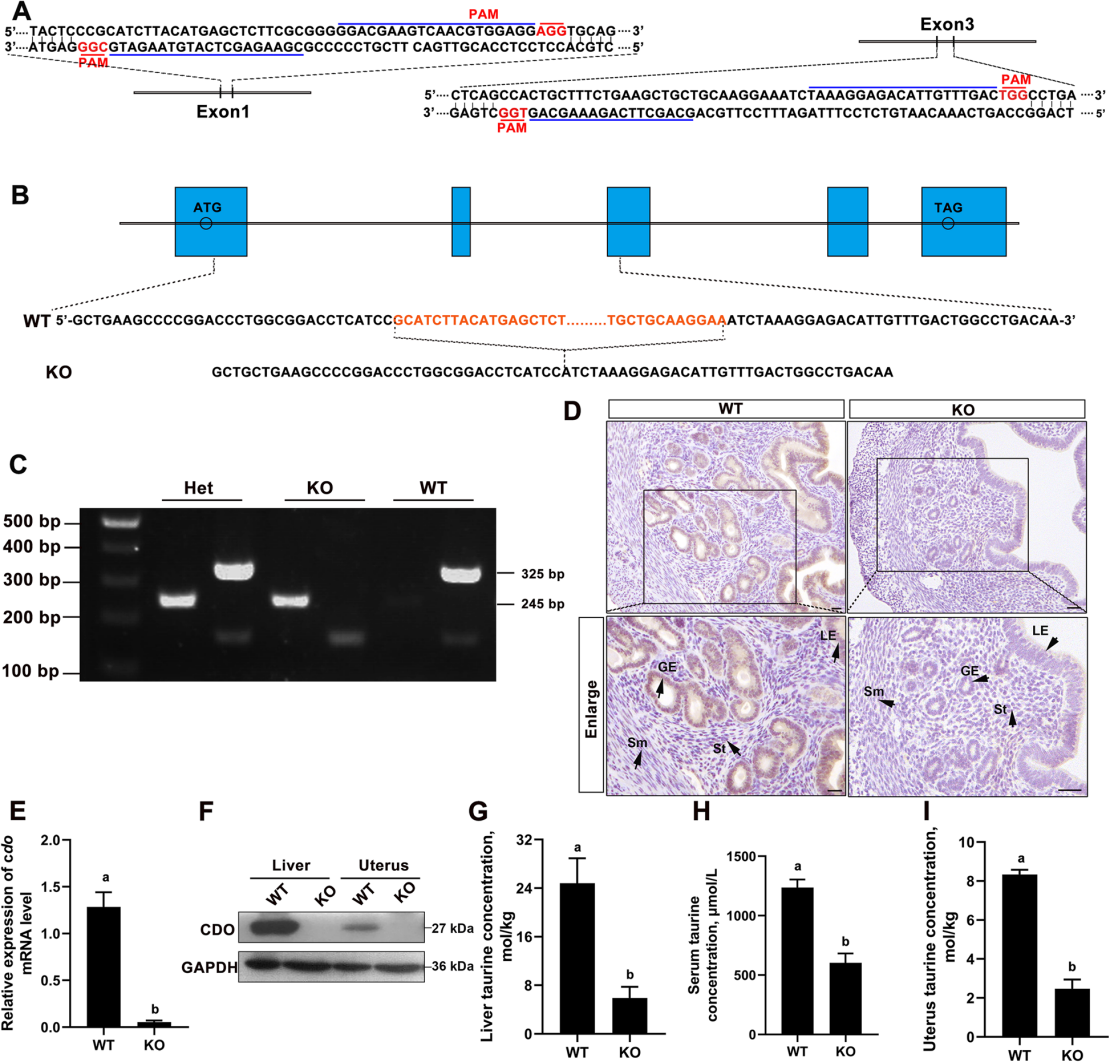
Generation of *Cdo* KO mouse. **A**, Cas9n target scheme in the first and the third exons of *Cdo* gene. The blue lines indicate sgRNAs’ spacer and the red lines indicate PAM (protospacer adjacent motif). **B**, Sanger sequencing of the target region in F0 mice. The *Cdo* KO mice get a miss of 7489 nucleotides. **C**, Genotype identification results using PCR. The 325 bp strand indicates wild type and the 245 bp strand indicates mutant type. **D**, IHC staining of CDO in WT and *Cdo* KO mouse uterus. Bars, 50 μm. **E**, RT-qPCR analysis of *Cdo* mRNA levels in WT and *Cdo* KO mouse uteri. **F**, Representative patterns of WB analysis of CDO expressions. **G**-**I**, Taurine levels in WT and *Cdo* KO mice liver, serum and uterus detected by HPLC. Data are the Mean ± SEM. (*n* ≥ 3). Different letters above columns indicate significant differences (*P* < 0.05). GE: glandular epithelium. LE: luminal epithelium. Sm: smooth muscle cells. St: stromal cells. Scale bars: 50 μm

To detect the functions of CDO in uterus, *Cdo* KO mice were generated by CRISPR/Cas9 technology. Two pairs of single guide RNAs (sgRNAs) (Table S1) targeting exon1 and exon3 of *Cdo* gene respectively (Fig. 2 A), and SpCas9n (D10A) mRNA were co-injected into d 1 embryos (Additional file 1). A *Cdo* KO mutant line with 7489 nucleotide deletion from the first to the third exon was used in the following experiments (Fig. 2 B). The genotypes were identified by PCR that the 245-base pair (bp) strand represent mutant type and the 325 bp strand represent wild type (Fig. 2 C). The knockout efficiency was identified by RT-qPCR, IHC and WB, and the results showed that CDO was totally deleted, at least in mouse uterus (Fig. 2D–F). As CDO is a key enzyme for taurine synthesis, we detected the changes of taurine levels after *Cdo* knocked out using HPLC. The results showed that the taurine concentrations in the liver, serum and uterus of *Cdo* KO mice decreased by 76.24%, 51.20% and 70.33% respectively than that of WT mice (Fig. 2G–I). These results demonstrate that CDO is successfully deleted from mouse genome and the lack of CDO leads to taurine deficiency.

### *Cdo* KO causes impairment of embryo implantation and severe subfertility

**Fig. 3:**
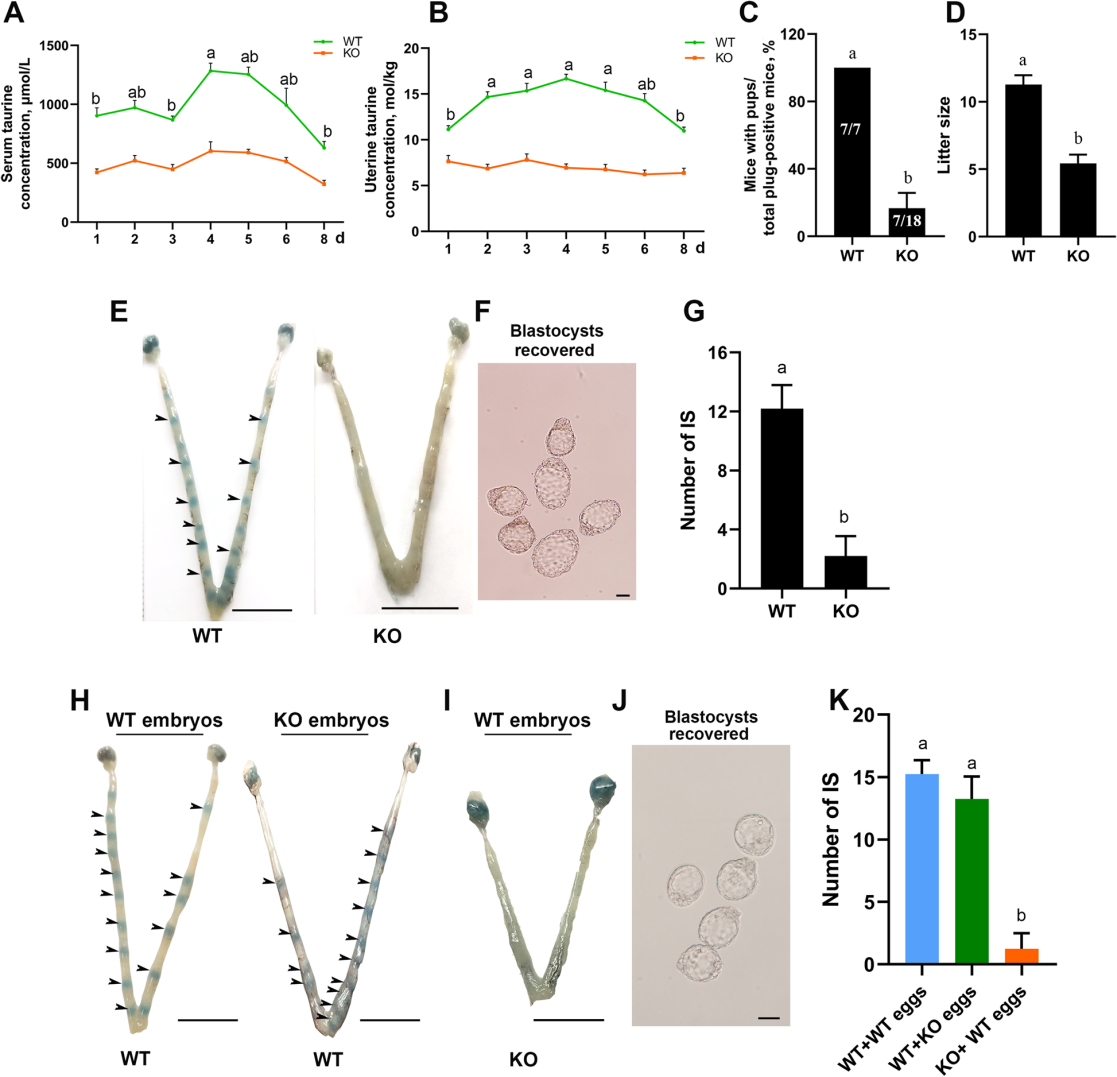
CDO deletion causes impairment of embryo implantation and severe subfertility. **A** and **B**, Serum (A) and uterus (B) taurine concentrations of pregnancy *Cdo* KO and WT females from d 1 to d 8, assayed by HPLC (*n* = 3). **C**, Pregnancy rates of WT and KO females. The number within brackets indicate females with pups over total number of plug-positive females. **D**, Average litter sizes of WT and *Cdo* KO females (*n* = 7). **E**, Representative images of d 5 pregnant uteri from WT and *Cdo* KO females. Bars, 1 cm. **F**, Blastocysts flushed from *Cdo* KO uterus. Bar, 50 μm. **G**, Average numbers of implanted embryos represented by implantation sites (*n* = 5). **H**, Representative patterns of d 5 WT receptive mice uteri transferred with WT and *Cdo* KO embryos. **I**, d 5 KO receptive mice uteri transferred with WT embryos. Arrowheads indicate the location of blastocysts. Bars, 1 cm. **K**, IS numbers of different treated mice uteri (*n* = 3). Data are presented as Mean ± SEM. Different letters above columns indicate significant differences (*P* < 0.05)

In order to identify the effects of *Cdo* deletion on the embryo implantation, the serum and uterine taurine levels of the *Cdo* KO females from d 1 to d 8 were assayed by HPLC. The results showed that *Cdo* KO sharply decreased the taurine levels in the serum and uterus tissue compared with that of WT mice, and there were no significant differences from d 1 to d 8 (Fig. 3A, B). However, there was a rising spike on d 4 in WT mice (Fig. 3A, B).

Further, the effects of *Cdo* deletion on the implantation and fertility were examined. Adult WT and *Cdo* KO females were respectively mated with ICR males, the parturition and pups of each litter were then recorded. The results showed that the plug-positive WT females exhibited normal fecundity, but only 38.8% (7/18) of the plug-positive *Cdo* KO females produced litters (Fig. 3 C), and the litter size (5.429 ± 0.65 pups/litter, *n* = 7) was much smaller than that of the WT females (11.29 ± 0.68 pups/litter, *n* = 7) (Fig. 3 D).

In order to answer whether the subfertility of *Cdo* KO mice was resulted from the abnormalities in the ovaries or embryos, the embryos were flushed out from oviducts of d 2 *Cdo* KO females, which naturally mated with WT males. The flushed 2-cell embryo numbers did not have significant difference between WT and *Cdo* KO mice (Fig. S2 B), and morphological defect was not observed either (Fig. S2 A). In addition, the histological abnormality was not observed in 10 weeks *Cdo* KO mouse ovaries (Fig. S2 C). Serum P_4_ and E_2_ levels did not exhibit significant differences between the *Cdo* KO and WT females on d 4 (Fig. S2 D and E).

Further, in order to dissect the cause of the subfertility in *Cdo* KO females, the effect of *Cdo* deletion on the embryo implantation was examined. The plug positive mice were euthanized on d 5 and the numbers of embryos implanted to the uterine epithelium were identified by employing the Chicago blue dye assay [39]. The implantation sites (IS) were clearly observed in WT females, but only a few or no IS were detected in *Cdo* KO females (Fig. 3 E, G). However, *Cdo* KO females had blastocysts which could be flushed out from uterus (Fig. 3 F).

In order to confirm whether the subfertility of *Cdo* KO females was resulted from the defects of uterus receptive status, *Cdo* KO and WT females were separately mated with *Cdo* KO and WT males to obtain *Cdo* KO and WT embryos. The harvested *Cdo* KO and WT embryos were respectively transferred to WT female uteri. On d 5, IS numbers were assayed and there was no significant difference between the WT females to which the WT and *Cdo* KO embryos were respectively transferred (Fig. 3 H, K). However, the IS number in *Cdo* KO receptive females was much less than in WT receptive females after both of which accepted WT embryos (Fig. 3 I, K). Meanwhile, blastocysts could be flushed out from *Cdo* KO uterus (Fig. 3 J). These infer that the failure of embryo implantation and subfertility in *Cdo* KO females are resulted from the defects of uterine receptivity.

### *Cdo* KO results in the defects of uterine receptivity by dysregulating PR and ER signaling in mouse

**Fig. 4:**
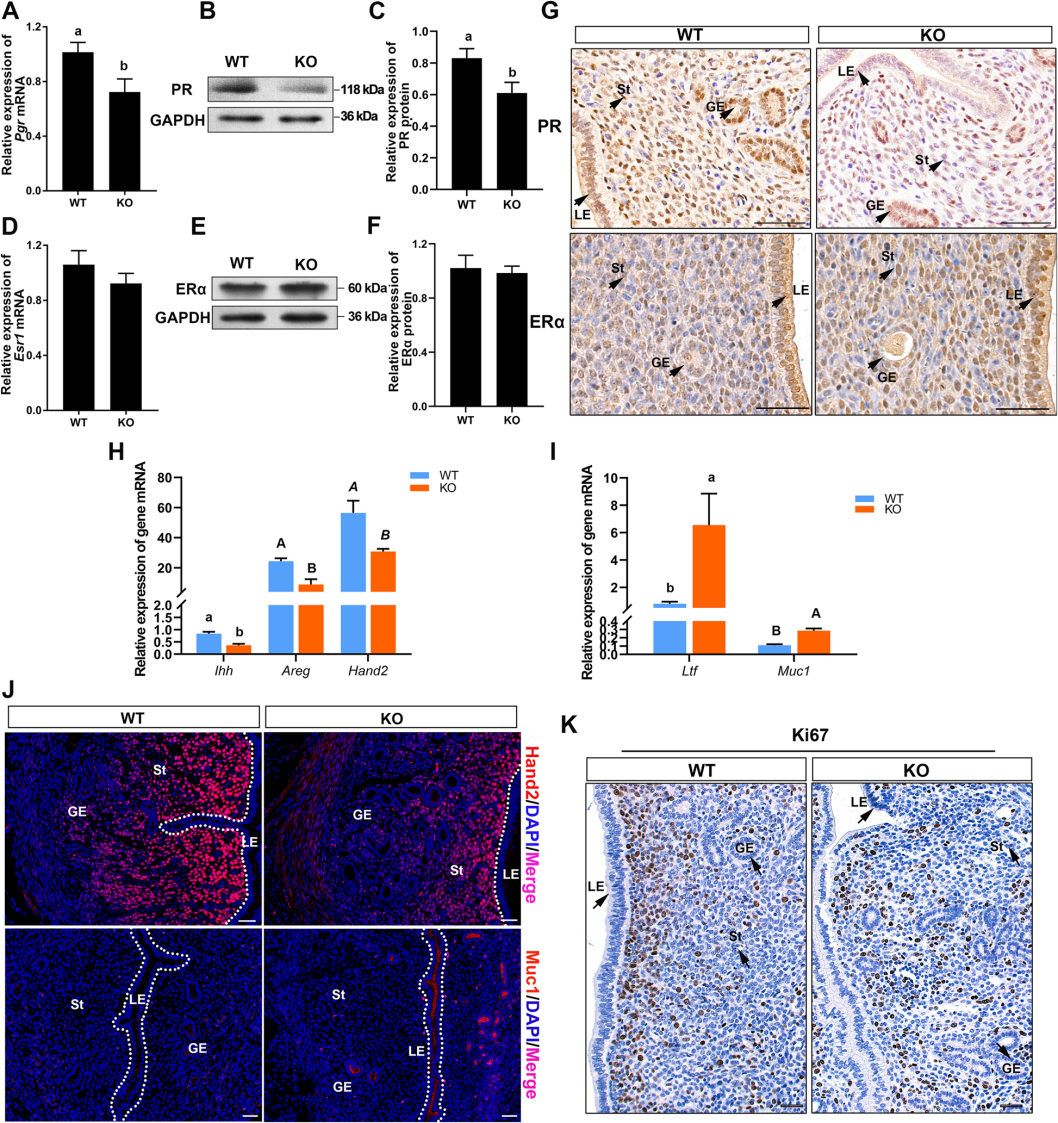
Effects of *Cdo* deletion on PR and ER signaling transduction and the cell proliferation in the uteri of d 4 pregnancy mice. **A**, RT-qPCR analysis of relative *Pgr* mRNA expression levels. **B** and **C**, WB detection and analysis of PR protein expression. **D**, RT-qPCR analysis of relative *Esr1* mRNA levels. **E** and **F**, WB detection and analysis of ERα protein expression levels. **G**, IHC staining of PR and ERα. Scale bars: 50 μm. **H**, Relative mRNA expressions of *Ihh, Areg* and *Hand2*. **I**, Relative mRNA levels of *Ltf* and *Muc1*. **J**, IF staining of Hand2 and Muc1. Scale bars: 50 μm. **K**, Ki67 (proliferative cell marker) IHC staining. Scale bars: 50 μm. GE: glandular epithelium. LE: luminal epithelium. St: stroma. Data are presented as Mean ± SEM, *n* ≥ 4. Different letters represent significant differences (*P* < 0.05)

Our results showed that *Cdo* KO had no significant effects on ovary morphology and functions, including E_2_ and P_4_ secretions (Fig. S2C–E). As the window of uterine receptivity coincides with the P_4_-mediated down-regulation of ERα activity in uterine LE, we thus assayed the effects of *Cdo* deletion on uterus PR expression on d 4 by RT-qPCR and WB. The results showed that PR mRNA and protein levels in *Cdo* KO uteri respectively decreased by 28.74% and 26.63% compared with that in WT uteri (Fig. 4A–C). IHC results showed that PR staining intensity on LE, GE and stromal cells were much weaker in *Cdo* KO mice than in WT mice (Fig. 4 G). In addition, the uterine mRNA expression levels of the known PR responsive genes *Ihh, Hoxa10* and *Hand2* decreased by 56.33%, 35.49% and 45.38% in KO mice than that of the WT mice (Fig. 4 H). Further, Hand2 IF staining was performed and it was observed that Hand2 was located only in the stroma cells, but its staining intensity was much weaker in *Cdo* KO mice than that in WT mice (Fig. 4 J), which was consistent with PR IHC staining result (Fig. 4 G).

In addition, *Cdo* deletion had no significant effects on ERα mRNA and protein levels (Fig. 4D–G). Whereas the expression levels of E_2_-responsive genes *Muc1* and *Ltf* elevated over 2.5 and 8 times in *Cdo* KO uterus than that of the WT uterus on d 4 (Fig. 4 I). The further IF staining confirmed the Muc1 expression in LE and GE cells of *Cdo* KO mouse uteri, but which was not detected in WT mice (Fig. 4 J).

Another important character of uterus receptivity to embryo is a cessation of epithelial cell proliferation and robust proliferation of stroma prior to implantation. We thus detected the cell proliferation in the uterine tissues of *Cdo* KO and WT mice on d 4 by Ki67 IHC staining. The results showed that Ki67 was not detected in LE and GE cells of WT and *Cdo* KO mice uterus, but Ki67 staining intensity in the stroma of WT uteri was much stronger than that of *Cdo* KO uteri. Moreover, Ki67 positive cells were concentrated around the LE in WT uteri (Fig. 4 K), but Ki67 positive cells in *Cdo* KO uteri were irregularly scattered (Fig. 4 K). These indicate that *Cdo* KO results in the defects of uterine receptivity by dysregulating PR and ER signaling in mouse.

### P_4_ and E_2_ are involved in regulating uterine CDO expression

**Fig. 5:**
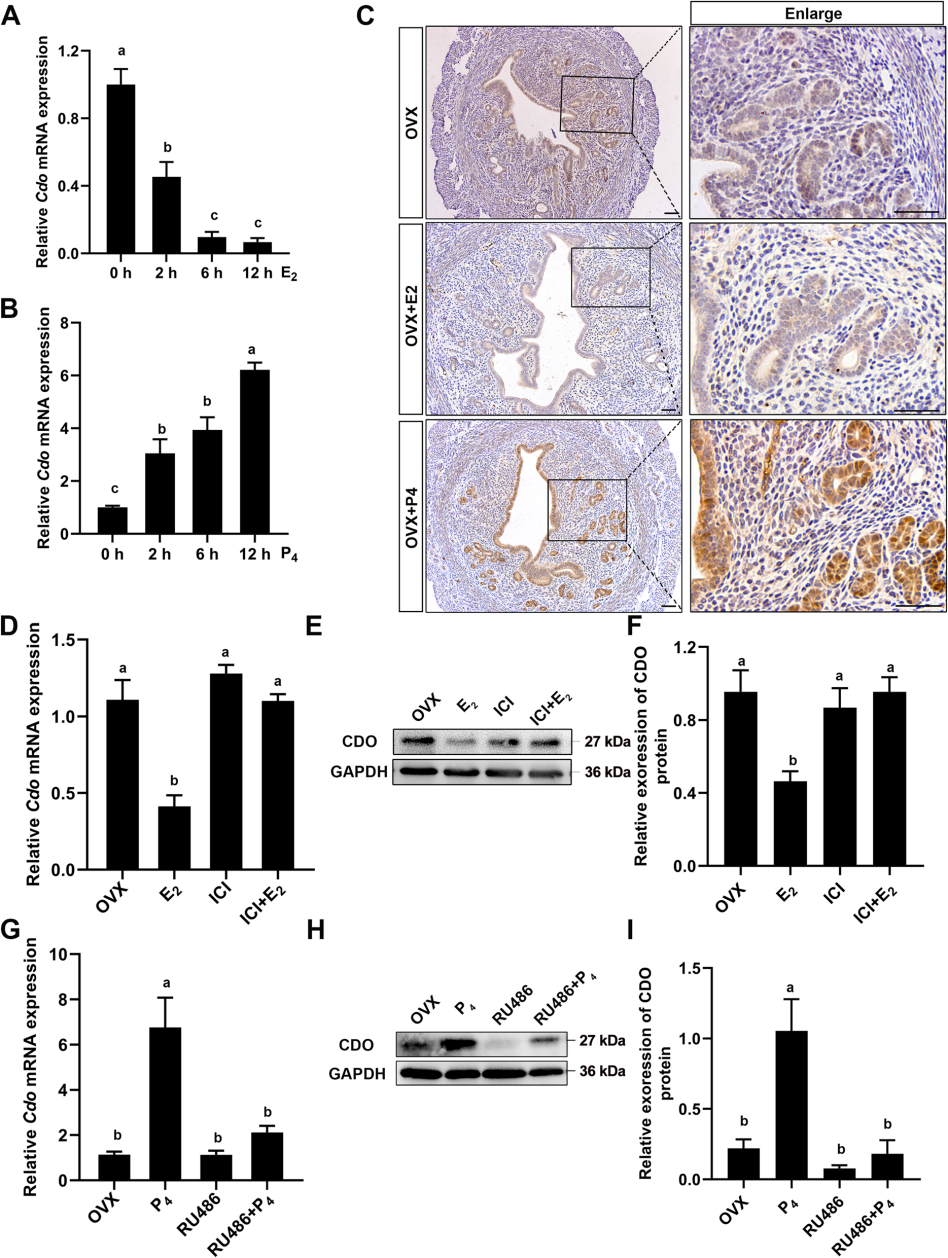
The relations of P_4_-PR and E_2_-ERα signaling to uterine CDO expression. **A** and **B**, Relative *Cdo* mRNA levels in OVX mouse uteri after 0 (control), 2, 6 and 12 h treatments with 100 ng E_2_ (**A**) and 2 mg P_4_ (**B**). **C**, CDO IHC staining in OVX mouse uteri after 0 (control) and 12 h 100 ng E_2_ or 2 mg P_4_ treatments. Scale bars: 50 μm. **D**, **E** and **F**, Relative *Cdo* mRNA (**D**) and protein levels (**E** and **F**) in OVX mouse uteri treated with E_2_ and E_2_ inhibitor ICI182780 (ICI). **G**, **H** and **I**, Relative *Cdo* mRNA (**G**) and protein levels (**H** and **I**) in OVX mouse uteri treated with 2 mg P_4_ and PR inhibitor RU486. Data are presented as Mean ± SEM. Different letters represent significant differences (*P* < 0.05, *n* = 3)

As the dynamic patterns of uterine CDO expression and taurine level were parallel to serum P_4_ concentration and uterine PR expression during early stage of pregnancy (Fig. 1A–D and Fig. 3A, B), we further identified the effects of P_4_ and E_2_ on uterine CDO expression by using OVX mouse model. RT-qPCR results showed that uterine *Cdo* mRNA levels were decreased by 54.65%, 90% and 93% after 2, 6 and 12 h after 100 ng E_2_ treatments in OVX mice (Fig. 5 A). These were further confirmed by the CDO IHC staining results that the CDO staining intensity got much weaker after 12 h E_2_ treatment (Fig. 5 C). It was opposite that 2 mg P_4_ injection sharply increased uterine CDO mRNA and protein levels, which were several folds higher than that of the controls after 12 h P_4_ treatment (Fig. 5 B and C). Further, in order to identify whether the regulating effects of P_4_ and E_2_ on uterine CDO expressions were through P_4_-PR and E_2_-ERα signaling, OVX mice were respectively pretreated with PR inhibitor RU486 and ERα inhibitor ICI182780, followed by P_4_ or E_2_ treatment. The uterine CDO expressions were then assayed and the results showed that RU486 blocked the enhancing effect of P_4_ on CDO expression (Fig. 5G–I), and ICI182780 restrained the inhibiting effect of E_2_ on CDO expression (Fig. 5D–F).

### Taurine supplement partly recues the defects of embryo implantation and subfertility caused by *Cdo* KO

**Fig. 6:**
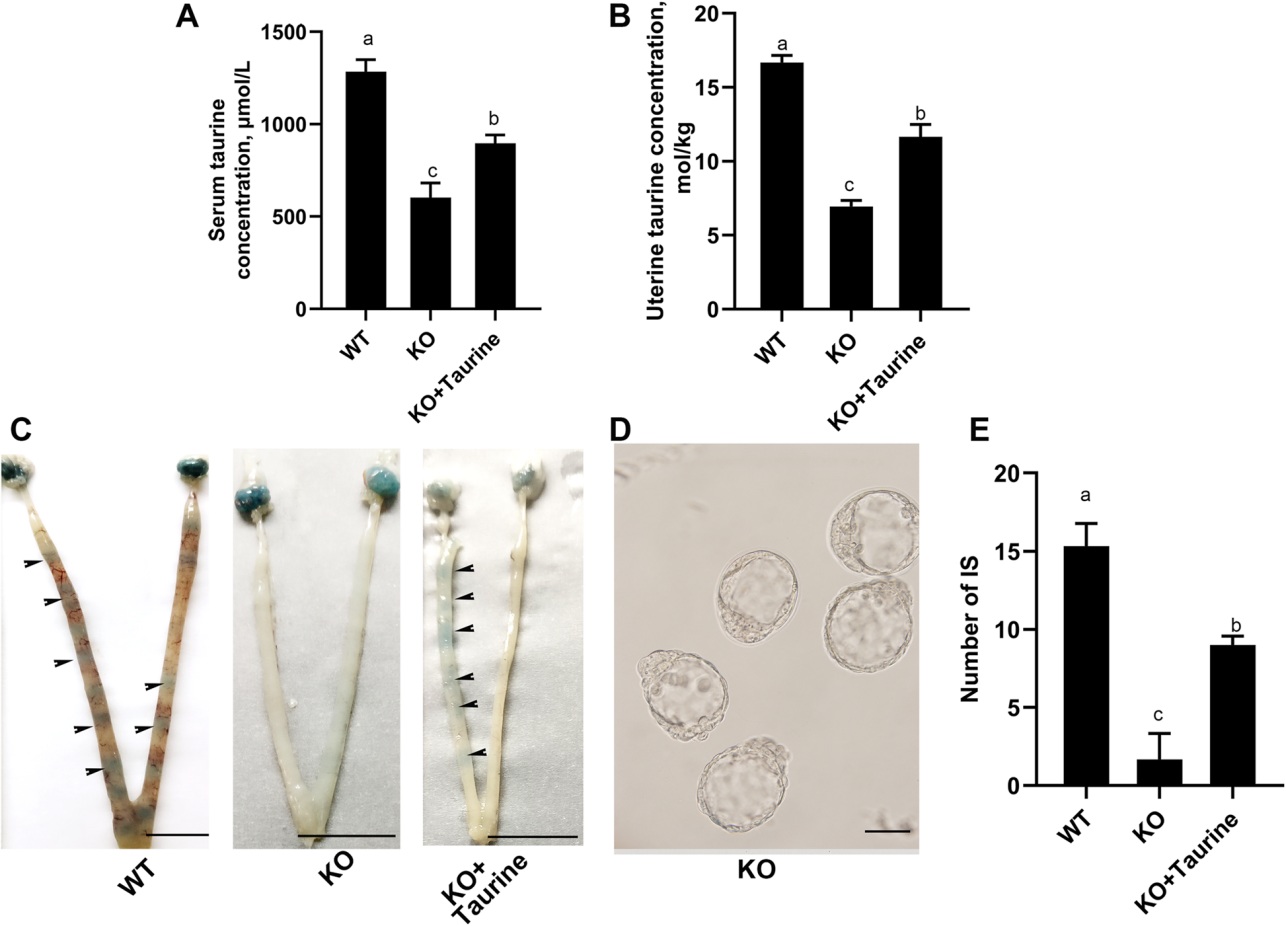
Taurine supplement increases the taurine levels in serum and uteri, and partly rescues the defects of embryo implantation in the CDO KO mice. **A**, Serum taurine concentration. **B**, Taurine concentration in uterus tissues. **C**, Representative images of implantation sites. Bars: 1 cm. WT: wide type female mice. KO: *Cdo* KO female mice. KO + taurine: *Cdo* KO female mice supplied with taurine. *n* = 3 for each genotype. **D**, Blastocysts flushed out from *Cdo* KO uterus. Bar, 50 μm. **E**, Numbers of implantation sites. Data are presented as Mean ± SEM. Different letters represent significant differences (*P* < 0.05, *n* ≥ 3)

As the maintenance of the global taurine level relays on the endogenous taurine synthesis through the action of CDO and the active uptake from the diet, we thus supplied extra taurine in the regular diet of *Cdo* KO mice to confirm whether the defects of uterine receptivity and subfertility caused by *Cdo* KO could be rescued. Adult female *Cdo* KO mice were supplemented with drinking water containing 0.2% (w/v) taurine. The uterine and serum taurine levels were assayed. The results showed that taurine supplement markedly elevated taurine levels in uteri and serum, but all of which were significantly lower than that in wild mice with regular diet (Fig. 6A, B). In addition, the embryo implantation was detected on d 5. The results showed that taurine supplement significantly increased the IS number of *Cdo* KO females, although it was still lower than that of the WT females (Fig. 6C–E). These results demonstrate that taurine supplementation can partly rescue the subfertility of *Cdo* KO females and suggest that CDO and taurine are essential factors for embryo implantation.

## Discussion

The present study, for the first time as we know, demonstrates that CDO and taurine play important roles for embryo implantation in mouse. This is supported by our results here that uterine CDO expression and serum taurine level during early stage of mouse pregnancy are parallel to the uterine taurine level, which sharply increases and reaches a peak just on d 4, the window of implantation [42], followed by a steadily decline under physiological condition. In addition, serum P_4_, a key factor to regulate the embryo implantation [7, 43], behaves a similar dynamic pattern with the uterine CDO expression and taurine concentration in the duration examined. Whereas global *Cdo* KO markedly decreases uterine taurine level and omits the dynamic pattern of uterine taurine, accompanied by the decline of serum taurine level, although *Cdo* KO does not affect the ovary functions to synthesize P_4_ and E_2_. These demonstrate that uterus has function to synthesize taurine, whereas *Cdo* deletion causes a severe subfertility in female mice. CDO and endogenous taurine synthesis are thus essential for embryo implantation and the maintenance of animal reproduction. These are also supported by our results here that taurine supplement to the *Cdo* KO mice can partly rescue the reproductive capacity.

Another important finding of this study is that *Cdo* KO causes defects in uterine epithelium receptivity and decline of pregnancy rate. It has been well documented that successful embryo implantation depends on the intimate connection between the blastocyst and maternal endometrial surface [43, 44]. Healthy embryos at the blastocyst stage and receptive maternal endometrium are necessary for the implantation [45]. The results of this study show that *Cdo* deleted embryos do not have morphological defects, and behave normal implantation as that of the WT embryos in WT recipients. However, the implantation and birth rates are dramatically decreased after the WT embryos are transferred to the *Cdo* deleted recipients. These imply that *Cdo* deleted embryos have normal capacity to get implantation. Another important character of uterine receptivity is the cessation in epithelial cell proliferation and robust proliferation in stroma cells just prior to implantation [46, 47]. The results of present study are in agreement with reports [44, 46] that the cell proliferation, marked by Ki67 IHC staining, is undetectable in the uterine epithelium, but the number of Ki67 positive stroma cells significantly decreases in *Cdo* KO mice compared with that of WT mice. These collective data suggest that *Cdo* deletion leads to the abnormalities of uterine receptivity and embryo implantation, and the subfertility of *Cdo* KO mice is primarily of uterine origin.

However, it has been reported that *Cdo* KO females are fertile and carry their pregnancies to term [26], whereas our results here demonstrate that 38.8% of plug-positive *Cdo* KO females are fertile, and the IS number and litter size of *Cdo* KO females are much less than that of WT females. These discordant statements might be resulted from the limited assessment, and no description about the statistics analysis of the IS numbers and birth rates by Ueki et al. [26]. Another possible explanation is that different mouse strains have been used in our study and the previous report [26].

In addition, the results presented here demonstrate that *Cdo* deletion impairs embryo implantation by dysregulating P_4_ and E_2_ signaling in mouse uterus. The related molecular and genetic studies indicate that both E_2_ and P_4_ play functions through their corresponding receptor ERα and PR [48, 49] together with local transcriptional and paracrine factors to govern the complicated embryo-uterine crosstalk [11, 12, 43, 50]. It is much interested that our results here show that CDO involves in regulating embryo implantation by affecting P_4_ and E_2_ signaling. In support, the uterus PR mRNA and protein levels in *Cdo* deleted mice are significantly decreased, accompanied by decline of *Ihh*, *Hoxa10* and *Hand2* expressions, which are known PR targeting molecules [18, 43, 51]. These are also confirmed by PR and Hand2 IHC results of this study. However, *Cdo* deletion does not affect ERα mRNA and protein levels, but the mRNA levels of E_2_-ERα responsive genes *Lf* and *Muc1* [14, 15] are increased over 5 and 1.5 time in *Cdo* KO mouse uteri, especially the abnormal expression of Muc1 in *Cdo* KO LE and GE cells. In addition, CDO or/and taurine might participate in E_2_-ERα signaling by affecting the ERα activity through ERα Ser 118 site phosphorylation [43, 52], although we did not detect it in this study. Collectively, the results presented here demonstrate that CDO is a crucial factor to affect uterine receptivity during embryo implantation by involving in P_4_-PR and E_2_-ERα signaling in mouse uterus, although the relating signaling pathways and mechanisms need to be clarified in future study.

Furthermore, the regulating effects of P_4_ and E_2_ on uterine CDO are confirmed by using OVX mouse model. The in vivo results show that uterus CDO expression level is decreased over 90% after 12 h E_2_ treatment in OVX mouse, which is in agreement with the reports that E_2_ inhibits taurine synthesis through estrogen-ERs-CDO/CSAD signaling in liver and uterus [37, 38]. It is inversed that uterine CDO expression level is sharply elevated after P_4_ injection in OVX mouse. In addition, it is well known that E_2_ and P_4_ are key drivers of uterine plasticity throughout the sexual cycle and early stage of pregnancy, we thus propose that both E_2_ and P_4_ may play roles through their respective receptors to regulate uterine CDO expression and taurine synthesis, which subsequently affect the uterine receptivity and embryo implantation. However, it remains to be elucidated about the interactions or cross-talks among P_4_-PR signaling, E_2_-ERα signaling, CDO and taurine in the duration of embryo implantation.

Finally, the maintenance of taurine levels in uterus and serum relays on the endogenous taurine synthesis through the action of CSAD, CDO, and the active uptake from the diet [34, 35], while *Cdo* deletion impairs embryo implantation and causes severe subfertility as it is showed in this study. Whereas taurine supplementation significantly increases the litter size and parturition rate of *Cdo* KO females (Table S2). These provide the in vivo evidence that CDO and taurine are crucial factors for the maintenance and improvement of animal production, and suggest that taurine may be potential agent for animal production.

In conclusion, the present study demonstrates that taurine and CDO play prominent roles for uterine receptivity and embryo implantation by involving in E_2_-ERα and P_4_-PR signaling pathways. These are crucial for our understanding the mechanism of embryo implantation, and infer that taurine is a potential agent for improving reproductive efficiency of livestock industry and/or for reproductive medicine. But it still remains to be elucidated about the interactions among P_4_-PR signaling, E_2_-ERα signaling, CDO and taurine in the duration of embryo implantation.

## Conclusions

The present study demonstrates that taurine and CDO play prominent roles for the uterine receptivity and embryo implantation by involving in E_2_-ERα and P_4_-PR signaling in early pregnancy stage. These elucidate a new mechanism for taurine regulating embryo implantation. On the other side, abuse of steroid hormones in stockbreeding industry usually gives rise to premature ovary failure and declines animal fertility. The balancing effect of CDO and taurine between P_4_-PR signaling and E_2_-ERα signaling shows that taurine is a potential agent for improving reproductive efficiency of livestock industry and reproductive medicine.

## Supplementary Information

**Additional file 1: Data 1.** Generation of *Cdo* knockout (KO) mice.

**Additional file 2: Table S1.** sgRNA pairs used with SpCas9n to target *Cdo* gene.

**Additional file 3: Fig. S1.** CDO mRNA and protein expressions in different tissues of female mice.

**Additional file 4: Fig. S2.** *Cdo* KO mouse ovary has normal morphology and functions.

**Additional file 5: Table S2.** Taurine supplement on *Cdo* KO female mice fertility.

**Additional file 6: Table S3.** Primer sequences for RT-qPCR.

**Additional file 7: Table S4.** Primer sequences for mice genotype identification.

## Data Availability

The datasets supporting the conclusions of this article are available from the corresponding author upon reasonable request.

## Abbreviations

CDO: Cysteine dioxygenase
E_2_: Estrogen
*Esr1* (ERα): Estrogen receptor 1
GE: Glandular epithelium
HA: Hyaluronidase
IF: Immunofluorescence
IHC: Immunohistochemistry
LE: Luminal epithelium
OVX: Ovariectomy
P_4_: Progesterone
*Pgr * (PR): Progesterone receptor
RIA: Radioimmunoassay
Sm: Smooth muscle cells
St: Stromal cells
WB: Western blotting
