# Supplementary material for: Cysteine dioxygenase and taurine are essential for embryo implantation by involving in E2-ERα and P4-PR signaling in mouse
Source: J Anim Sci Biotechnol. 2023 Jan 5;14:6. doi: 10.1186/s40104-022-00804-1 (PMC9814424; doi:10.1186/s40104-022-00804-1)
Supplement: Supplementary file 1 — Additional file 1: Data 1. Generation of Cdo knockout (KO) mice. [file 40104_2022_804_MOESM1_ESM.docx]

**Data 1 Generation of *Cdo* knockout (KO) mice**

*Cdo* KO mice were generated by CRISPR/Cas9. SpCas9n (D10A) (Addgene plasmid # 48873) was used in this study as reported by Ran et al. [1]. Two pairs of guide sequences in codon region of the first exon and in the third exon of mouse *Cdo* gene (Table S1) were selected, which have no potential off targets (http://crispr.mit.edu). The guide sequence oligoes were synthesized by Sangon Biotech Co., Ltd (Shanghai, China). The sgRNAs and SpCas9n mRNA were synthesized as reported [2, 3]. Gene KO mice were generated by micro-injection the mixture of SpCas9n (D10A) mRNA (60 ng/μL) and sgRNAs (25 ng/μL) into cytoplasm of d 1 embryos and then transferred to the oviduct of a pseudopregnancy females. The F 0 generation mice were genotyped by PCR (Primer sequences listed in Table S4) and Sanger sequencing (Sangon, Shanghai, China) of target region using genomic DNA extracted from tails. *Cdo* heterozygous (*Cdo* Het) mice were crossed to generate *Cdo* KO mice.

**References**

1. Ran FA, Hsu PD, Wright J, Agarwala V, Scott DA. Zhang F. Genome engineering using the CRISPR-Cas9 system*.* Nat Protoc. 2013;8:2281–308. <https://doi.org/10.1038/nprot.2013.143>.

2. Li JF, Norville JE, Aach J, McCormack M, Zhang D, Bush J, et al. Multiplex and homologous recombination-mediated genome editing in Arabidopsis and Nicotiana benthamiana using guide RNA and Cas9*.* Nat Biotechnol. 2013;31(8):688–91. <https://doi.org/10.1038/nbt.2654>.

3. Wang H, Yang H, Shivalila CS, Dawlaty MM, Cheng AW, Zhang F, et al. One-step generation of mice carrying mutations in multiple genes by CRISPR/Cas-mediated genome engineering*.* Cell. 2013;153(4):910–8. <https://doi.org/10.1016/j.cell.2013.04.025>.
