# Supplementary material for: Cysteine dioxygenase and taurine are essential for embryo implantation by involving in E2-ERα and P4-PR signaling in mouse
Source: J Anim Sci Biotechnol. 2023 Jan 5;14:6. doi: 10.1186/s40104-022-00804-1 (PMC9814424; doi:10.1186/s40104-022-00804-1)
Supplement: Supplementary file 3 — Additional file 3: Fig. S1. CDO mRNA and protein expressions in different tissues of female mice. [file 40104_2022_804_MOESM3_ESM.docx]

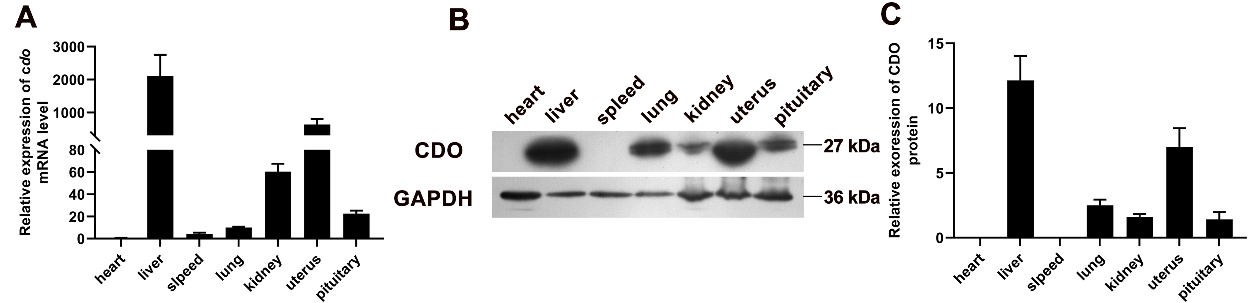
**Fig. S1** CDO mRNA and protein expressions in different tissues of female mice. **A**, *Cdo* mRNA levels assayed by RT-qPCR. **B** and **C**, CDO protein levels detected by WB. Results are presented as Mean ± SEM (*n* ≥ 3)
