# Supplementary material for: Cysteine dioxygenase and taurine are essential for embryo implantation by involving in E2-ERα and P4-PR signaling in mouse
Source: J Anim Sci Biotechnol. 2023 Jan 5;14:6. doi: 10.1186/s40104-022-00804-1 (PMC9814424; doi:10.1186/s40104-022-00804-1)
Supplement: Supplementary file 4 — Additional file 4: Fig. S2. Cdo KO mouse ovary has normal morphology and functions. [file 40104_2022_804_MOESM4_ESM.docx]

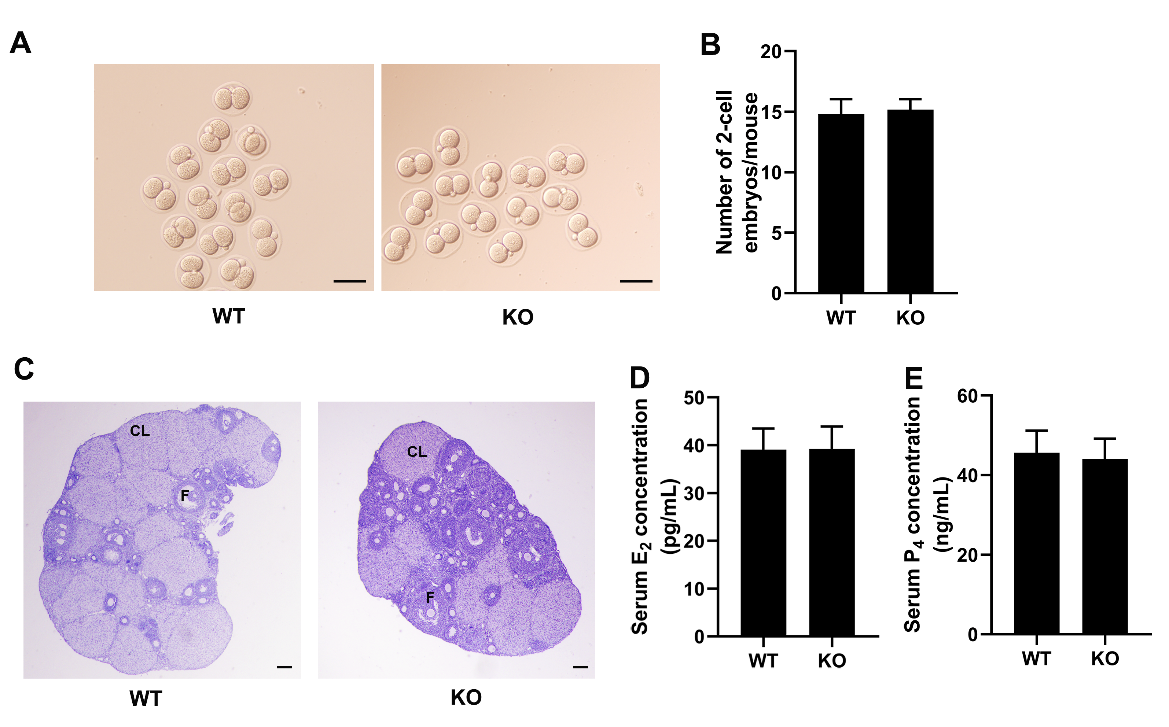


**Fig S2.** *Cdo* KO mouse ovary has normal morphology and functions. **A**, 2-cells obtained from naturally mated female mice. Bars: 50 μm. **B**, The numbers of 2-cell embryos obtained from WT and *Cdo* KO females (*n* ≥ 5). **C**, Hematoxylin-eosin staining of 10 w mice ovaries. Bars: 50 μm. **D** and **E**, Serum E_2_ and P_4_ concentration detected by RIA (*n* = 4). Data are shown as Mean ± SEM. CL, corpus luteum; F, follicle
